# Quality as part of universal health coverage in Bhutan: a narrative synthesis

**DOI:** 10.1016/j.lansea.2026.100758

**Published:** 2026-04-02

**Authors:** Jayendra Sharma, Milena Pavlova, Wim Groot

**Affiliations:** Department of Health Services Research, CAPHRI, Maastricht University Medical Center, Faculty of Health, Medicine and Life Sciences, Maastricht University, P.O. Box 616, Maastricht, 6200, MD, the Netherlands

**Keywords:** Bhutan, Health, Health system, Quality, Universal health coverage

## Abstract

Quality of care is fundamental for countries’ progress towards universal health coverage (UHC). While access to health care and financial protection have received substantial attention in both global- and country-level discussions and monitoring systems, attention to quality of care in the context of UHC has remained rather limited. Through a combination of document analysis and narrative synthesis approach, this study describes and synthesizes the existing evidence on quality of care in Bhutan, in the context of its progress toward UHC. The results suggest that while the population health outcomes have improved, there are significant shortfalls in care competencies, and a need to strengthen the quality foundations through system-wide action. Specific attention needs to be paid to key quality imperatives such as strengthening governance mechanisms, enhancing clinical competency with measurable quality indicators, streamlining patient flows in primary and specialist care, and addressing the urgent gaps in the health care workforce.

## Introduction

Improving quality of care is fundamental for countries’ progress towards universal health coverage (UHC) and the Sustainable Development Goals (SDG).^1^^,^^2^ The global monitoring framework for UHC stipulates that health services need to be of “sufficient quality to be effective.”^3^ The SDG target 3·8 calls for achieving “universal health coverage, including financial risk protection, access to quality essential health care services and access to safe, effective, quality and affordable essential medicines and vaccines for all”.^4^ Quality is key to UHC, because even if essential health coverage and financial protection is ensured, health outcomes would still be poor if services are of low quality and unsafe.^5^ Therefore, improving quality of care needs to be firmly embedded within the framework of UHC.

While access to health care and financial protection have been central to UHC policy dialogues and monitoring frameworks, the issue of health care quality has lagged in prominence and attention.^6^^,^^7^ Despite several global and national initiatives on health care quality, the weak integration of quality in UHC constrains reform discussions and monitoring efforts. Quality plays a critical role in equitable health services. If the quality of care received is poor or below standard and fails to consider the diverse needs across populations, this may impede equality in treatment outcomes.^8^ For instance, those who can afford better quality care in the private sector may opt out of the public health care system.

Evidence shows that quality of care in many countries is suboptimal.^5^ Between 5·7 million and 8·4 million people in low- and middle-income countries die each year because of poor quality of care, representing 15% of all deaths in these countries.^1^ Although there is insufficient evidence on the quality-mortality nexus, the global losses due to low health care quality have been estimated to amount to US$6 trillion in economic losses in 2015 alone.^2^

Improvement in health care delivery requires a deliberate focus on the quality of health services, which involves providing effective, safe, people-centered care that is timely, equitable, integrated, and efficient.^7^ High-quality health systems need to drive progress on UHC. A high-quality health system is defined as one “that optimizes health care in a given context by consistently delivering care that improves or maintains health outcomes, by being valued and trusted by all people, and by responding to changing population needs.”^2^

There are, however, challenges in anchoring quality within the UHC framework. A recent scoping review, canvassing 45 studies spanning multiple geographical regions, confirmed that quality of care was often ill-defined or defined inconsistently, compromising the development of a robust evidence base to inform quality-related policies and interventions.^6^ Quality is viewed as a secondary priority to more primordial issues such as availability, service delivery, and financing in the Western Pacific region.^7^ In the South Asia region, poor governance of the health sector contributes to low-quality health care in most countries.^9^ Such gaps in conceptual positioning and monitoring could limit policy attention to quality, hindering meaningful progress towards UHC. Achieving UHC built on a firm foundation of safe and high-quality care, together with all that is necessary to sustain it, is the current imperative in health policy.^5^

**Fig. 1:**
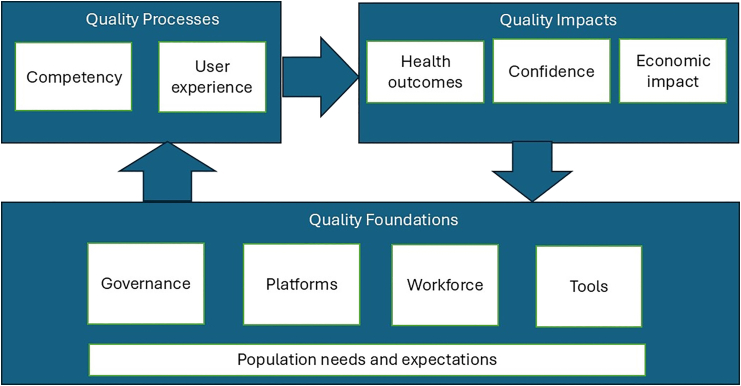
High-quality health system. Adapted from Kruk's high-quality health system framework.^2^

Very few studies have attempted to provide a comprehensive country-level assessment of quality of care anchored in the health system framework. We based our review on the conceptual framework for high-quality health systems,^2^ which shares the closest relevance to our research objectives (Fig. 1). The conceptual framework adopts a holistic health systems approach and suggests three key domains for high-quality health systems: foundations, processes of care, and quality impacts. This framework builds on Donabedian's conceptual model of quality of care, and important methodological developments thereafter to offer a quality measure for health systems over time on elements that matter to people (processes and impacts) and to guide opportunities for improvement (foundations).^2^

The framework was used as follows: first, the key domains from the framework guided the thematic grouping and extraction of key messages from the selected literature. Second, considering the scope of this study, we mildly adapted the framework with the following two changes in its illustration: (1) we used neutral phrases to name the domains in quality processes and quality impacts considering the bi-directional potential of the domains, and (2) we adjusted the positioning of “population needs and expectations” to infer relevance to all domains under quality foundations.

We focus on Bhutan, a lower-middle-income country located between China and India, with a population of 784,043 dispersed across a mountainous and topographically challenging landscape. The health system is predominantly financed and delivered through the public sector. Health services are structured across three tiers: primary care through outreach clinics, sub-posts and primary health centres; secondary care through district hospitals; and tertiary care provided by national and regional referral hospitals. Despite consistent policy efforts to address quality and the institution of several quality improvement initiatives, quality of care continues to be a challenge. A national-level comprehensive health system review shows that monitoring of quality and safety in health services requires a significant push.^10^ Complaints about long waiting times and the demand for better quality and client-friendly services are increasing, particularly in urban settings and among the more affluent population groups.^10^ To meet increasing consumer expectations for high-quality health care services, the growing burden of chronic diseases, and aligned with the policy emphasis on Gross National Happiness (GNH), Bhutan needs to invest in patient-centered health systems that deliver high-quality services.^10^^,^^11^ The objectives of the study are to describe, analyze and synthesize the existing literature on quality of care in Bhutan, in the context of its progress toward UHC. The study aims to identify evidence needed to support UHC discussions and highlight directions for policy in Bhutan, while contributing to methodological discussions in low- and middle-income countries’ contexts.

### Search strategy

Our approach can best be described as a document analysis (review of policy reports and guidelines) combined with a narrative synthesis (review of research publications).^12^, ^13^, ^14^ Considering the heterogeneity of data sources and the value of an integrative synthesis, we explored multiple sources of data.

1. We searched PubMed for literature in English that was published in the ten years up to 10 May 2025. MeSH terms “bhutan” and “health” were applied along with the keyword “quality”. Search terms and their possible synonyms and spellings were identified and used in the search string available in Supplementary Table S1.
2. We identified and scanned through the websites of the government and development partners active in the country to retrieve any publications on health systems and quality in Bhutan in the last ten years.

We adopted an iterative approach where evidence on all the available concepts, rather than on studies, is sought until saturation is reached.^15^ To identify additional studies, we snowballed from the above sources or identified them through keyword searches to canvass the scope of the study till a redundancy point was achieved. Inclusion criteria applied in the selection of records were articles published in the last 10 years on Bhutan that addressed health system and/or quality improvement efforts. We excluded studies that did not address at least one component of the framework (Fig. 1).

A total of 27 records were selected. The PRISMA 2020 flow diagram^16^ and the profile of the selected literature is available as Supplementary Fig. S1 and Table S2. The Enhancing Transparency in Reporting the Synthesis of Qualitative Research (ENTREQ) statement^15^ was used to ensure transparency and adequate reporting of the review. The checklist and the list of selected publications are available as Supplementary Tables S3 and S4. Considering the heterogeneous sources of publications, an appraisal of the research quality of the publications was not carried out.

### Quality impact

This review indicates sustained improvements in population health outcomes in Bhutan in the past four decades. The GNH Index, a composite national measure of socio-economic progress, has improved over the years, from 0·743 in 2010, 0·756 in 2015 and 0·781 in 2022.^17^ Health is firmly embedded and is the top contributor (13·9%) among the total of nine domains of GNH.^17^ With life expectancy at birth at 69·5 years, Bhutan recorded sex-specific life expectancy gains of 23–29 years in the past 40 years, situating it among the global best performers in the increase in life expectancy in the last four decades.^10^ The UHC service coverage index shows an improving trend, increasing from 44 in 2010 to 60 in 2021. This is enabled by positive outcomes in infectious disease control and progress in maternal and child health.^18^ Mortality rates have been significantly reduced since the development of the modern health system in the 1950s. As per the most recent health survey, crude death rate stood at 10·2 deaths per 1000 population, Maternal Mortality Ratio was 53 deaths per 100,000 live births, infant mortality rate was 15·2, and the under-five mortality rate was 19·5 per 1000 live births.^19^ However, much less is known about the quality of care and outcomes for non-communicable diseases, which are growing in prominence and account for 69% of all deaths.^10^

The evidence points toward a high level of user confidence and trust in the health system in Bhutan, although the trend has been worsening in recent years. A large majority (91·2%) of people visiting health facilities reported satisfaction with the health services, with women, poorer households and rural residents reporting higher levels of satisfaction.^19^ A similar proportion (96·1%) indicated that they would return for service at the same facility and 97·5% of patients would recommend the facility at which they received services on the day of the survey to a family or friend.^20^ However, the proportion of the population not satisfied with health services increased from 6·8% in 2012 to 8·2% in 2023.^19^ Lack of specialized care remains the most prominent reason for patients choosing multiple points of care.^10^^,^^20^

Suboptimal quality continues to exert a substantial macroeconomic burden in Bhutan and poses increasing financial hardship to the population. The value of lost welfare due to amenable mortality represented 10·6% of Bhutan's gross domestic product (GDP) in 2015.^21^ In 2019, the direct and indirect economic losses due to tobacco use was estimated at Ngultrum 1·2 billion (approximately 15 million US dollars) equivalent to 0·7% of its annual GDP.^22^ Similarly, Bhutan incurs high economic costs of alcohol, with alcohol-related treatment estimated at 1·8% of current health expenditure and socio-economic cost of around Ngultrum five billion (approximately 60 million US dollars) incurred annually.^23^ Alcohol liver disease was the leading cause of deaths reported by health facilities in 2022 and 2023.^24^ While the association is not directly established, shortcomings in quality of health care continue to weigh heavily on Bhutan's economy.

Total health care costs at Bhutan's referral hospitals and district level hospitals have increased in real terms by 62% and 54% respectively between 2009–10 and 2019–20.^25^ Inefficiencies and wastage in the health system is poorly documented. Free-of-charge medicines and supplies create limited cost-consciousness and value for services among the population, often leading to a waste of health resources.^10^ Catastrophic health expenditure and impoverishment increased between 2007 and 2017, with cumulative financial hardship affecting 2·55% of the population in 2017 with increased burden on the poor, larger and vulnerable households.^26^

### Quality processes

The review highlights notable gaps and variations in care competencies across treatment categories and health facility levels in Bhutan. A survey among all health facilities, using clinical vignettes, found that provider diagnostic and treatment accuracy is high for acute child health conditions, but low for stunting and anemia.^20^ Similarly, provider knowledge in the comprehensive management of intrapartum emergencies was reported as inadequate.^20^ A recent national health survey estimated that 13% of patient with hypertension on treatment did not have their blood pressure controlled.^19^ Among women who were more than 20 weeks pregnant, only 60·6% reported having received all of the set of five key antenatal care services.^20^ Obstetric complications, intrapartum complications and prematurity were identified as primary determinants of facility-based neonatal mortality in Bhutan, underscoring the importance of quality antenatal care.^27^

Medication errors, hospital-acquired infections, surgical errors and postoperative complications, diagnostic errors, laboratory/blood errors, fall injuries, communication errors and patient identification errors were perceived as key patient safety concerns in Bhutan, driven by both system and human factors.^28^ Health care professionals rated “staffing and work pace” and “reporting patient safety events” as the weakest dimension of patient safety culture in Bhutan.^29^ Event reporting, communication openness and handoffs and transitions require immediate attention to improve patient safety practices in hospitals.^30^ A clinical audit report on nursing documentation at the national hospital revealed that 48·9% of nursing records did not have the nursing care process documented.^31^ With the majority of the providers feeling overworked, Bhutanese hospitals have an average patient safety culture and there are gaps that need urgent attention.^29^

In addition, adherence to standard care protocols varied considerably across health facilities. Documentation of prescribed or dispensed antibiotics was only available in 71·1% of health facilities, while 60·6% conducted testing for nationally notifiable diseases.^20^ Only 49·1% maintained routine records for point-of-care cleaning, and a mere 8·8% had at least one staff member trained in antimicrobial resistance or antibiotic stewardship within the past two years.^20^ Availability of emergency preparedness protocols and guidelines is notably deficient in primary health facilities, despite their crucial role in serving local populations during emergencies.^20^

The selected studies attribute negative user experiences to over-crowding of hospitals, waiting time and access to specialized care. Bhutanese patients rated health care facilities/providers high on the domains of communication, autonomy and respect during their visits to health facilities, while rating low on waiting time and travel time to reach health facilities.^20^ Waiting time experiences vary across health facilities, increasing with the level of the health facilities, with a median of 5 min at primary and secondary health facilities and 20 min at the tertiary level health facilities.^20^ Overcrowding of secondary and tertiary hospitals due to poor gatekeeping mechanisms at the primary health care centers has been recognized as a significant issue.^10^^,^^32^ Patients receiving care at primary facilities reported the highest rates of referrals to another facility, with 89% of these referrals attributed to the availability of specialized services, equipment, medicine, and supplies.^20^

### Quality foundations

The literature highlights a strong commitment to quality health care embedded in Bhutan's health system, predominantly financed and delivered by the government. This governance structure is anchored in the Constitutional provision to ensure “free access to basic public health in both modern and traditional medicine”.^10^ The Ministry of Health stewards the health system together with the clinical oversight body named the National Medical Services and decentralized public health entities at the district level. The Bhutan Food and Drug Authority regulates the medical products, food and biosecurity systems, while the Bhutan Qualifications and Professionals Certification Authority regulates health professionals. While private sector participation in health care has historically been limited to retail pharmacies, there has been a gradual increase in the presence of private diagnostic centers in major towns and an increasing number of Bhutanese using health care abroad in the last few decades.^26^ Significant inroads into leveraging multisectoral partnerships and collaboration for the health sector were demonstrated during the “whole of government, whole of society” approach to COVID-19, as well as the new thirteenth five-year plan “social sector cluster” governance approach.^33^

The national health policy and strategic plans emphasize the prioritization of quality enhancement in health care.^32^^,^^33^ The Quality Assurance and Standardization Division was established in 2002 “to institute an effective quality management system in all health care centres, ensure continuous quality improvement and innovative health management practices.”^33^^,^^34^ Structured quality improvement tools have been instituted, such as the Hospital Administration and Management Transformation tool and the Bhutan HealthCare Standard for Quality Assurance (BHSQA). The BHSQA consists of 106 standards, 639 objective elements and 68 key performance indicators based on national and global quality standards.^33^ In addition, there are several quality improvement initiatives that demonstrate the potential of simple and cost-effective interventions to improve the quality of care in health facilities.^35^^,^^36^

Fragmented responsibilities and coordination challenges have been observed,^30^ potentially hindering the development of a cohesive and strategic governance and leadership framework. Gaps in the implementation of BHSQA at the primary health care level continue to hinder quality and patient safety initiatives.^33^ There appears to be a lack of urgency and accountability, and patient safety has been overlooked by leaders and managers, with efforts mostly confined to isolated projects and personal initiatives.^28^^,^^37^

Despite the rapid scale-up of health services in Bhutan, the review identifies ongoing difficulties in streamlining patient movement. Since the onset of planned socio-economic development in mid-1950s, the country has witnessed a significant increase in health infrastructure, which currently consists of 55 hospitals with a total of 1660 inpatient beds, 187 primary health centers and 557 outreach clinics.^10^^,^^24^ At the primary level, health infrastructure comprises primary health centers, satellite and outreach clinics. Secondary level services are provided by district level hospitals, where specialized services are provided by three regional referral hospitals. Almost three out of four households (72·9%) reported living within a 30-min travel time from the nearest health facility (hospitals or primary health centers).^19^ However, it is widely acknowledged that a weak enforcement of the referral structure results in the by-passing of primary health care services and overcrowding of major hospitals.^10^^,^^33^^,^^38^^,^^39^

Bhutan continues to face persistent challenges in human resource availability and distribution, particularly due to its small and limited pool of physicians.^33^ Significant vacancies exist in health facilities with an acute shortage of and capacity gaps, particularly noteworthy at the specialized levels.^10^ In addition, a recent trend of increased health worker attrition, with a larger number of Bhutanese working abroad, could further exacerbate human resource challenges.^33^ A survey among selected health facilities across primary, secondary, and tertiary care levels revealed high workload pressure corresponding to high staffing gaps in these health facilities.^40^ In addition, while supportive supervision mechanisms for the health workforce that promotes facilitative professional supervision and mentoring are established at central and local levels, the implementation is weak.^33^ Urgent interventions are needed to bolster capacities and retain existing staff.

The availability of essential drugs and supplies in Bhutan is consistently high, but facilities in rural and remote areas face medicine shortages.^20^^,^^33^ There are also variations in the availability of essential equipment and diagnostic supplies across health facilities, particularly those in remote and rural areas.^33^ The Ministry of Health monitors the quality of medical products and equipment, though it is often constrained by resources to address urgent maintenance requests from rural and far-flung areas.^10^ For instance, in a spot check of blood pressure measuring devices in health facilities, just about 64·72% passed the pressure accuracy test, and 71·85% passed the allowable leak rate.^41^

Bhutan's health management information system has improved rapidly over the last several decades. Over 95% of health facilities in Bhutan reported using District Health Information System (DHIS)-2, which was consistently high across regions and facility types.^33^ The strong investments in the nationwide integrated health information system, along with the ongoing major initiative on the electronic patient information system, hold strong potential to significantly enhance historical personal care records and generate real-time evidence for health sector decisions.^33^

The review documents increasing health literacy and quality consciousness among the population along with a continued influence of deeply rooted cultural practices. Health literacy has significantly improved over the years owing to general improvements in population literacy rates, rigorous health promotion activities and high-level public health advocacy efforts.^10^ This combined growth in health-seeking behaviour and the expansion of health facilities in the last four decades has brought the population closer to health services. In 2023, 78·6% of the population aged 15–69 years old visited health facilities for any health concern, with a majority (55·7%) identifying hospitals as their nearest point of access.^19^ With increasing health literacy, quality consciousness, and the transitions to electoral politics since 2008, demand for quality and more convenient care is rapidly growing.^10^^,^^11^ A distinctive aspect of this landscape is the strong cultural emphasis on religious rituals for health and wellbeing, locally known as *rimdo* or *puja*. Bhutanese households spent a similar amount as their out-of-pocket spending on health care for these activities.^10^ Household survey data from 2017 indicate that 31% of individuals who reported illness or injury did not seek modern medical care and people living in rural areas had 3·4 times higher odds of using primary health centers compared to outpatient hospital care.^39^

## Discussion

This review aimed to profile health system quality in Bhutan, in the context of the country's progress toward UHC. Several key shortfalls in the domains of quality foundations and quality processes emerged from this review, similar to other countries in the South Asia region.^9^^,^^42^, ^43^, ^44^, ^45^, ^46^

Notwithstanding the enabling health legislation and policies that express Bhutan's commitment to a high-quality health system identified in this review, there are gaps in effective leadership and a robust feedback loop on quality of care at various levels of the health system. Leadership styles and practices have a major impact on quality, patient outcomes, health care workforce, and organizational culture.^47^ It is essential that quality improvement initiatives do not emerge out of individual or project-based initiatives but are integrated into the leadership and management culture across all levels of the health system. The review uncovered a wide range of fragmented quality improvement programmes and initiatives in Bhutan led by the Ministry of Health, health facilities, and some individual initiatives in their own professional circuits. Such fragmentations could contribute to the sub-optimal impact of these initiatives. There are substantial opportunities to improve coordination and the implementation of quality improvement at a more strategic level; mobilizing stakeholders and expertise and supporting implementation across levels of the health system.

Our study highlights a significant gap in the quality process, marked by deficiencies and variations in care competencies across treatment categories and health facility levels. Prevention and early detection of non-communicable diseases are strong quality markers of programmatic success because they reduce long-term disease burden and lower health care costs. Competent care must define the whole health system as well as individual care visits, and people should be able to count on their conditions being detected and managed in an integrated manner.^2^ The health system needs to ensure that individual care visits result in evidence-based and appropriate treatment, counseling, and management based on a correct diagnosis. This would entail more structured care pathways with competencies enabled through robust and accountable clinical care processes. The Lancet Global Health Commission on high-quality health systems recommends establishing a national quality guarantee for health services, which specifies the level of competence and user experience that people can expect.^2^

Despite significant expansion in access to health services, this review identifies ongoing difficulties in achieving optimum patient flow at different levels of health facilities. Overcrowding of major hospitals, largely concentrated in the western region, and a weak referral system that leads to by-passing of primary health care services are important concerns.^10^^,^^33^^,^^38^ The health sector needs to improve first-contact utilization and coordination of primary care as well as referral to specialist care. The recent advances in the adoption of digital health tools have been noteworthy; a fully functional electronic patient information system based on the International Classification of Diseases (ICD)-11th Revision and the institution of the annual household health surveillance system^48^ will meaningfully enhance data standardization, disease classification and quality monitoring, if the momentum is effectively sustained.

As highlighted in this study, the health workforce gaps will undermine progress toward high-quality UHC in Bhutan. Despite notable efforts by the government, health workforce challenges continue to manifest as staff shortages, especially in the specialist cadre, as well as maldistribution in human resources among districts and between urban and rural areas.^10^^,^^33^ The high rates of attrition among health care workers in recent years reflect gaps in welfare or satisfaction of health professionals, besides the economic reasons and ambitions. These challenges have the potential to undermine UHC progress. Despite the ongoing work on a competency framework for health professionals, which incorporates measurable benchmarks for clinical competencies, high levels of attrition among health care workers could translate to poor quality of services across all levels. Therefore, health workforce gaps should be addressed in a decisive manner, through consistent policies and long-term, predictable funding, while ensuring that the policies are fully aligned with national needs, strategies, and accountability mechanisms.^49^

The multi-dimensional factors influencing quality require the availability and use of a broad array of data and empirical evidence that could pose a challenge in a low-resource context. In addition, evidence is fragmented and does not provide a complete overview of quality of care in the country. A high-quality health system requires accurate, reliable and timely health Information.^7^^,^^50^ Information enhances accountability, which in turn improves quality.^2^^,^^7^ This implies that the health system needs to integrate quality metrics into country monitoring and information systems through a manageable and resourced set of indicators and data collection tools to help advance progress towards high-quality UHC. This includes identifying and monitoring service delivery points and population groups experiencing poor quality of care.

The study reveals gaps in the strategic outlook for quality anchored on UHC goals, while quality impacts permeated both dimensions of UHC; coverage of essential services and financial protection. This resonates with the widespread concern across the globe on the weak emphasis and linkages between quality and UHC.^2^^,^^6^^,^^7^^,^^51^ This is in addition to the complexities involved in understanding quality of care, often inadequately covered by existing definitions and measurement approaches.^50^ The framework of UHC Global Monitoring Report suggests capturing the dimension of quality through its indicators on “effective coverage.”^52^ However, given that this approach only covers the tracer coverage indicators of selected services, the overall health systems approach to quality is missing. The Lancet Global Health Commission on high-quality health systems reemphasizes that improving quality will require system-wide action and quality should be a core component of UHC.^2^ As countries continue to move towards UHC and implement the broader sustainable development agenda, the World Health Organization and other global partners will need to raise the profile of health system quality in their support to countries and in their normative guidance.^51^

Methodologically, this paper provides a case study for the application of Kruk's high-quality health systems framework^2^ to the discussions on UHC. The strengths of this framework lie in its application of a health system approach to address quality of care. We recognize that the framework has enabled a comprehensive perspective on health system quality for progress toward UHC. At the same time, the framework had some limitations in addressing the objectives of our study; the alignment to UHC monitoring framework is inadequate, and there are remaining concerns on data availability in low- and middle-income settings for country-level assessments.

The study has several limitations. First, the methodology is prone to potential bias in the selection, presentation and interpretation of evidence, leading to selected papers not representative of all relevant research and policy documents, including singular database search and exclusion of material from unpublished sources. Second, the heterogeneity across studies in terms of source and quality of evidence could have reduced transparency and replicability. We tried to cross-review the synthesis among authors to minimize bias. We have also used the ENTREQ reporting checklist to enhance transparency in reporting the results.

## Conclusion

This study highlights that while the population health outcomes have improved in Bhutan, there are significant shortfalls in care competencies and the need to strengthen the quality foundations through system-wide action. To further strengthen Bhutan's progress towards UHC, a critical starting point would be to embed quality of care within the country's UHC policies and monitoring framework while establishing a national guarantee for health service quality. Key policy priorities include strengthening governance mechanisms that integrate quality improvement efforts across all levels, enhancing clinical competency with measurable quality indicators, streamlining patient flow in primary and specialist care, and addressing the urgent gaps in health workforce.

The review also demonstrates the application of high-quality health systems framework for country-level assessment. Further efforts to raise the profile of health system quality anchored on UHC in global normative guidance and monitoring frameworks are critical. Country-level UHC policies and their monitoring and accountability frameworks need to integrate all dimensions of health system quality, encompassing quality impacts, processes and foundations. Future research is essential to guide this approach and develop practical and user-friendly toolkits to ensure meaningful progress toward UHC.

## Contributors

All authors contributed to the study conceptualization and design. JS led the material preparation, data collection and analysis. MP and WG accessed and verified the underlying data and analysis reported in the manuscript. JS wrote the first draft of the manuscript. All authors contributed to revision of the manuscript and approved the final version.

## Data sharing statement

All data generated or analyzed in this study are included in the manuscript and the supplementary files.

## Declaration of interests

Authors declare no competing interests.
