# Supplementary material for: Quality as part of universal health coverage in Bhutan: a narrative synthesis
Source: Lancet Reg Health Southeast Asia. 2026 Apr 2;48:100758. doi: 10.1016/j.lansea.2026.100758 (PMC13084747; doi:10.1016/j.lansea.2026.100758)
Supplement: Supplementary Tables and Figures [file mmc1.pdf]

Table of Contents

Supplementary Table S1. Database Search String....2

Supplementary Fig S1. Records identification flow chart....3

Supplementary Table S2. Search results: profile of the publications....4

Supplementary Table S3. Enhancing transparency in reporting the synthesis of qualitative research: the ENTREQ statement....5

Supplementary Table S4. Selected literature and theme addressed....7

Supplementary Table S1. Database search string

Database	Search string
Pubmed	("bhutan"[MeSH Terms] OR "bhutan"[All Fields] OR "bhutan s"[All Fields]) AND ("health"[MeSH Terms] OR "health"[All Fields] OR "health s"[All Fields] OR "health system"[All Fields] OR "healths"[All Fields]) AND ("quality"[All Fields] OR "qualities"[All Fields] OR "quality s"[All Fields])

Supplementary Fig S1. Records identification flow chart

The database search process retrieved 161 records, of which eight records were selected after de-duplication and title/abstract screening. In total, 12 government reports and documents were included. In addition, seven records were identified snowballing from the previous records and new keyword search to meet the scope of the study. This led to the inclusion of a total of 27 records (Fig. below). The selected studies are listed in Supplementary Table S3.

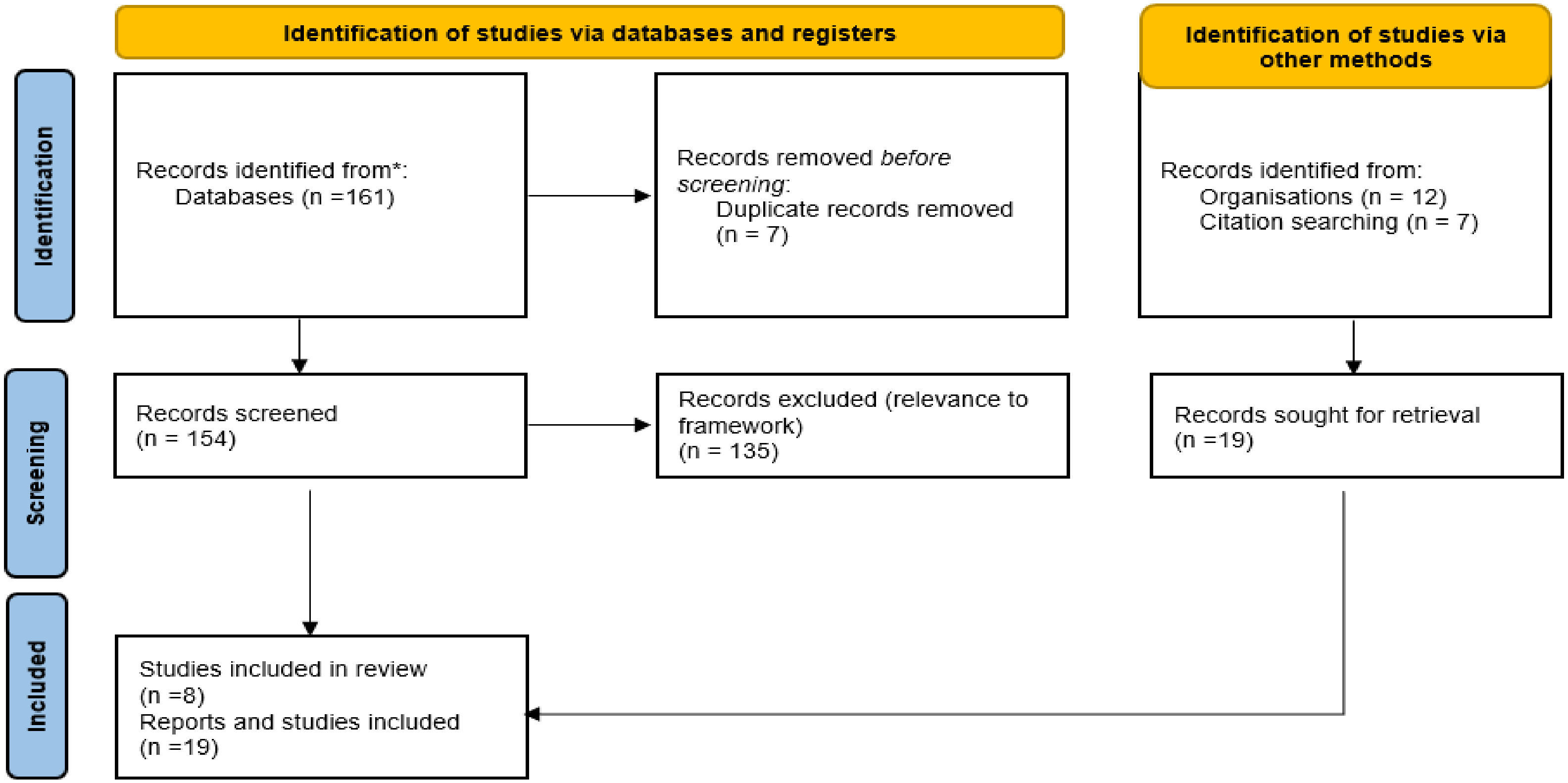

Supplementary Table S2. Search results: profile of the publications

Table (below) provides the general profile of the literature. A large component of evidence comes from published reports of the government and development partners. The number of publications has significantly increased in the last five years, demonstrating an increased level of research and policy interest in this subject. The domains being addressed by the literature do not have major variations, with the lowest number on "quality processes."

**Characteristics**	***N* (%)**
Publication year	
2015-2019	5 (18·5)
2020-2024	22 (81·5)
Publication type	
Report	12 (44·4)
Quantitative	11 (40·7)
Qualitative	3 (11·1)
Perspective	1 (3·7)
Frequency of themes addressed (multiple counts)	
Quality foundations	13 (36·1)
Quality processes	11 (30·6)
Quality impacts	12 (33·3)

Supplementary Table S3. Enhancing transparency in reporting the synthesis of qualitative research: the ENTREQ statement

**No.**	**Item**	**Guide and description**	**Orig. Pg #**
1	Aim	State the research question the synthesis addresses.	2
2	Synthesis methodology	Identify the synthesis methodology or theoretical framework which underpins the synthesis, and describe the rationale for choice of methodology *(e.g. meta-ethnography, thematic synthesis, critical interpretive synthesis, grounded theory synthesis, realist synthesis, meta-aggregation, meta-study, framework synthesis).*	2-3
3	Approach to searching	Indicate whether the search was pre-planned (*comprehensive search strategies to seek all available studies)* or iterative (*to seek all available concepts until they theoretical saturation is achieved).*	3
4	Inclusion criteria	Specify the inclusion/exclusion criteria *(e.g. in terms of population, language, year limits, type of publication, study type).*	3
5	Data sources	Describe the information sources used (e.g. *electronic databases (MEDLINE, EMBASE, CINAHL, psycINFO, Econlit), grey literature databases (digital thesis, policy reports), relevant organisational websites, experts, information specialists, generic web searches (Google Scholar) hand searching, reference lists)* and when the searches conducted; provide the rationale for using the data sources.	3
6	Electronic Search strategy	Describe the literature search *(e.g. provide electronic search strategies with population terms, clinical or health topic terms, experiential or social phenomena related terms, filters for qualitative research, and search limits).*	3
7	Study screening methods	Describe the process of study screening and sifting *(e.g. title, abstract and full text review, number of independent reviewers who screened studies).*	3
8	Study characteristics	Present the characteristics of the included studies *(e.g. year of publication, country, population, number of participants, data collection, methodology, analysis, research questions).*	5
9	Study selection results	Identify the number of studies screened and provide reasons for study exclusion *(e,g, for comprehensive searching, provide numbers of studies screened and reasons for exclusion*	5

		indicated in a figure/flowchart; for iterative searching describe reasons for study exclusion and inclusion based on modifications t the research question and/or contribution to theory development).	
10	Rationale for appraisal	Describe the rationale and approach used to appraise the included studies or selected findings *(e.g. assessment of conduct (validity and robustness), assessment of reporting (transparency), assessment of content and utility of the findings).*	3
11	Appraisal items	State the tools, frameworks and criteria used to appraise the studies or selected findings *(e.g. Existing tools: CASP, QARI, COREQ, Mays and Pope; reviewer developed tools; describe the domains assessed: research team, study design, data analysis and interpretations, reporting).*	NA
12	Appraisal process	Indicate whether the appraisal was conducted independently by more than one reviewer and if consensus was required.	3
13	Appraisal results	Present results of the quality assessment and indicate which articles, if any, were weighted/excluded based on the assessment and give the rationale.	NA
14	Data extraction	Indicate which sections of the primary studies were analysed and how were the data extracted from the primary studies? *(e.g. all text under the headings "results /conclusions" were extracted electronically and entered into a computer software).*	3
15	Software	State the computer software used, if any.	NA
16	Number of reviewers	Identify who was involved in coding and analysis.	NA
17	Coding	Describe the process for coding of data *(e.g. line by line coding to search for concepts).*	3
18	Study comparison	Describe how were comparisons made within and across studies *(e.g. subsequent studies were coded into pre-existing concepts, and new concepts were created when deemed necessary).*	NA
19	Derivation of themes	Explain whether the process of deriving the themes or constructs was inductive or deductive.	3
20	Quotations	Provide quotations from the primary studies to illustrate themes/constructs, and identify whether the quotations were participant quotations of the author's interpretation.	NA
21	Synthesis output	Present rich, compelling and useful results that go beyond a summary of the primary studies (e.g. *new interpretation, models of evidence, conceptual models, analytical framework, development of a new theory or construct).*	6-12

Supplementary Table S4. Selected literature and theme addressed

Sl #	Author, year	Title	Article type	Theme addressed		
				Quality foundations	Quality process	Quality impact
1	Thinley et al. 2017	Kingdom of Bhutan Health System Review; Health Systems in Transition	Report	✔	✔	✔
2	Ministry of Health, 2023	5th National Health Survey; Integrated Stepwise Household Survey 2023	Report	✔	✔	✔
3	World Bank, 2024	Service Delivery Indicators Health Survey for Bhutan 2022-2023	Report		✔	✔
4	World Bank, 2024	Primary Health Care in Bhutan; Findings from the Primary Health Care Performance Initiative Assessment	Report	✔	✔	✔
5	Ministry of Health, 2020	Annual Quality Assurance Report 2020	Report	✔		
6	Royal Government of Bhutan, 2024	Thirteenth Five Year Plan 2024-2029	Report	✔	✔	
7	Ministry of Health, World Bank and Global Fund, 2023	Bhutan Healthcare Costing Analysis 2023	Report			✔
8	Ministry of Health, 2024	Annual Health Bulletin 2024	Report			✔
9	Ura et al. 2022	GNH 2022	Report			✔
10	UNDP 2024	Investment Case for Tobacco Control in Bhutan	Report			✔

11	WHO South-East Asia Regional Office, 2024	Monitoring progress on universal health coverage and the health-related Sustainable Development Goals in the South-East Asia Region: 2024 update	Report			✔
12	Ministry of Health and World Health Organization, 2021	Workload Indicators of Staffing Need (WISN) in Selected Health Facilities of Bhutan	Report	✔		
13	Chhetri et al. 2022	Team approach to fall reduction in paediatric ward of national referral hospital, Thimphu, Bhutan: a quality improvement initiative	Quantitative	✔		
14	Wangmo et al. 2025	Improving drug charting practices and documentation among nurses in emergency department at a regional hospital, Bhutan: a quality improvement initiative	Quantitative	✔		
15	Pelzang and Hutchinson 2018	Patient safety issues and concerns in Bhutan's healthcare system: a qualitative exploratory descriptive study	Qualitative		✔	
16	Pelzang et al. 2017	Culture matters: indigenizing patient safety in Bhutan	Qualitative	✔		
17	Pelzang 2023	Why Bhutan needs a policy push on person-centred care	Qualitative		✔	
18	Sharma et al. 2024	Rural-urban inequalities in health care utilization in Bhutan: a decomposition analysis.	Quantitative	✔		
19	Lethro et al. 2023	Determinants of Neonatal Mortality in Bhutan: A Case-Control Study	Quantitative		✔	
20	Dorji et al. 2025	Patient Safety Culture in Bhutan	Quantitative		✔	

21	Sharma et al. 2023	Catastrophic health care expenditure and impoverishment in Bhutan	Quantitative			✔
22	Damrongplasit and Wangdi 2017	Healthcare utilization, bypass, and multiple visits: the case of Bhutan	Quantitative	✔		
23	Tamang et al. 2021	How reliable are the current Blood Pressure Measuring devices in Health Facilities of Bhutan?	Quantitative		✔	
24	Pelzang et al. 2023	Patient safety culture among healthcare professionals in Bhutan	Quantitative	✔	✔	
25	Zangmo et al. 2021	A clinical audit report on quality of nursing documentation at Jigme Dorji Wangchuck National Referral Hospital	Quantitative		✔	
26	Alkire et al. 2018	The Economic Consequences of Mortality Amenable to High-Quality Health Care in Low- And Middle-Income Countries	Quantitative			✔
27	Tshering et al, 2024	Improving Sustainable Financing for Universal Health Coverage in Bhutan: Exploring Policy Options and Financial Strategies	Perspective			✔
